# Outcomes of the Extreme Elderly Undergoing Anaesthesia and Surgery amongst Southeast Asians

**DOI:** 10.1155/2020/4562528

**Published:** 2020-03-30

**Authors:** Si Jia Lee, Oriana Ng, Sze Ying Thong

**Affiliations:** Singapore General Hospital, Outram Road, Singapore 169608

## Abstract

**Introduction:**

With a rapidly ageing population in Singapore, we see an increasing number of elderly patients undergoing surgery, both elective and emergency. This study aims to look at the anaesthesia techniques employed in a subset of very elderly population undergoing surgery and their subsequent postoperative outcomes, in particular their 30-day mortality, postoperative complication rates, and length of hospital stays.

**Materials and Methods:**

We searched from our hospital records between 2012 and 2013 for patients equal to or older than 90 years old who have undergone surgery and retrospectively analysed the types of surgery and mode of anaesthesia used.

**Results:**

Sixty-two patients were identified. The mean age is 93.6 years. Majority were ASA 2 and ASA 3 patients. The most common type of surgery performed was orthopaedic, followed by vascular and urologic. Seven of the 62 patients required re-operations. Regional was the predominant anaesthetic technique employed, followed by general anaesthesia. Intraoperative hypotension was seen in 16 of the patients, all of whom recovered uneventfully. Hypothermia, desaturation, and hypertension were the top three complications observed in the recovery. Seventeen patients were admitted to a high-dependency facility postoperatively. The mean length of stay was 13.7 days. The 30-day mortality was 1.6 percent.

**Conclusions:**

We have provided a snapshot of very elderly patients coming for surgery. The results show that this group of elderly patients do well postoperatively with relatively low complication and 30-day mortality rates. The outcomes presented can be used as a guide for risk counseling in the perioperative period.

## 1. Introduction

With an increasingly ageing population and rising life expectancy, more patients we see constitute the extreme elderly patients. Singapore is a country with one of the most rapidly ageing populations. In the year 2014, 0.7% of the population was more than 85 years old. By 2020, this will double to more than 1.7%. The average life expectancy of a male and female in Singapore has also steadily increased over the years to 79.9 and 84.5 years, respectively [1]. An ageing patient population poses a set of problems to anaesthetists, not only are these patients who live longer more likely to suffer from an accumulation of diseases during their lifetime there is also a constant deterioration in the various physiological systems. This makes them fragile medically; hence, anaesthesia and surgery on this select cohort were challenging. Our study aims to look at the anaesthetic techniques employed in the subset of very elderly population (≥90 years old) undergoing surgery and their postoperative outcomes.

## 2. Materials and Methods

Medical records of patients ≥90 years old who underwent any surgery requiring anaesthetic support between Jan 2012 and Feb 2013 were retrieved and retrospectively analysed from the hospital records of Singapore General Hospital. Institutional Review Board approval was obtained for the study.

Seventy-five patients were included under the criteria search, of which 62 patients were recruited in our study. Thirteen were excluded from the study due to incomplete or unavailable case notes. Seven of the 62 patients underwent repeat surgeries during the same admission. In these patients, data pertaining to their surgeries were taken from the first operation.

Some of the key clinical indices collected include age, gender, American Society of Anaesthesiologists (ASA) physical status, past medical history, basic laboratory findings, mode of anaesthesia, types of intraoperative monitoring, intraoperative events, and postoperative complications. A summary of these can be found in Tables 1–4.

Surgeries were classified as elective or emergency and in accordance with their disciplines. For the elective surgeries, patients were reviewed up to 1 month prior to the surgery for optimisation in the preoperative anaesthetic evaluation clinic (PAC). Those who were admitted for urgent or emergency surgeries were seen either in the wards when the primary team made an anaesthetic referral, or reviewed in the operating theatre (OT) reception prior to surgery. There are protocols in place, developed by PAC to guide workup of abnormal clinical findings (e.g., new murmurs) or deranged investigation results. Initiation of specialist referrals is also guided by specific department protocols, but can also be made at the primary anaesthetist's discretion. Cardiac-related protocols are developed based on the American College of Cardiology/American Heart Association (ACC/AHA) clinical practice guidelines [2].

All patients receiving anaesthesia were monitored in accordance with ASA monitoring standards, including the presence of a trained anaesthesia personnel at all times, continual heart rate, blood pressure monitoring (noninvasive or intra-arterial), and pulse oximetry [3] In addition, those receiving general anaesthesia (GA) or deep sedation have continual sampling of end-tidal carbon dioxide, inspired gases, and oxygen concentrations.

Patients who underwent GA were preoxygenated with oxygen, coinduced with fentanyl and propofol, and maintained on either sevoflurane or desflurane. Atracurium or rocuronium was used whenever muscle relaxation was indicated and reversed with neostigmine and glycopyrrolate. In the group that received regional anaesthesia (RA), they either had a centraneuraxial block or peripheral nerve block (PNB) with or without sedation. The sedation agents used comprised of propofol infusion, or intermittent boluses of midazolam and/or ketamine. For centraneuraxial blocks, the choice of local anaesthetic was 0.5% bupivacaine (+/− dextrose additive), with or without fentanyl. PNBs are performed under ultrasound guidance using a specialised echogenic nerve block needle (e.g., Stimuplex).

Patients were transferred to our postanaesthetic care unit (PACU) postoperatively and monitored for at least 30 minutes. The same continuous pulse oximetry, cardiac telemetry, and five-minute noninvasive blood pressure (NIBP) monitors were used in PACU, with a nurse to patient ratio of up to 1 : 2. All patients are discharged by the anaesthetic medical officer on duty in PACU, or the anaesthesia on-call team, in accordance with a locally designed discharge criteria modelled after the modified Aldrete scoring system [4].

Rescue analgesia was given in PACU, either in the form of oral acetaminophen or intravenous opioids (fentanyl, morphine, or pethidine). The postoperative complications were followed up to the point of discharge from the hospital (Table 3). Immediate complications that occurred in PACU were identified by reviewing the PACU charts, while subsequent complications in the rest of the hospital stay were captured through reviewing the patient's case notes and discharge summaries. Perioperative hypotension was defined as ≥20% drop in systolic blood pressure (BP) from the baseline (taken as the preinduction BP), for 2 or more readings. Hypothermia was defined as a core body temperature of ≤35°C and desaturation as SpO_2_ less than 95%. Readmissions to hospital due to surgical complications were retrieved from the electronic medical records system.

Data were analysed using the Statistical Package for the Social Sciences version 21.0 (SPSS Inc, Chicago, IL, USA). Categorical data were presented in the form of numbers and percentages. Continuous variables of normal distribution were presented as means and standard deviations (SD).

## 3. Results

### 3.1. Patient Demographics

The 62 patients identified had a mean age of 93.6 (±2.4) years.  Table 1 summarizes some of the pertinent patient demographics and surgical characteristics. The most common type of surgery performed was orthopaedic, followed by general surgery and then urologic. A vast majority of the operations performed (92.3%) were classified under minor and intermediate cardiac risk in accordance with ACC/AHA guidelines for cardiac risk stratification in noncardiac surgical procedures. Two-thirds of the operations were of emergency nature. Most of our patients were ASA 3 (71%) and ASA 2 (25.8%). Hypertension, anaemia, and diabetes mellitus were the top 3 most common medical conditions.

### 3.2. Preanaesthetic Assessment

A quarter of our patients (25.8%) received a cardiology review, while only 1 (1.8%) was referred for early anaesthetic review. Three of the 16 who were reviewed by cardiology preoperatively received some form of cardiac optimisation through initiation of beta-blockade for presumptive ischemic heart disease, BP optimisation, advice on antiplatelet cessation, and heart failure management. In one of them, an incidental finding of valvular vegetation was made on transthoracic echocardiography (TTE), resulting in rescheduling of the surgery. Amongst the 16 cardiology referrals, 13 of them were made solely for cardiac risk assessment and stratification. This comprised of a TTE and a clinical risk assessment by the cardiologist. Ten of these 13 referrals resulted in no further action recommended by the cardiologist. Except for one patient who underwent surgery on the same day as the referral, the other 15 patients saw delays in surgery ranging from 3 to 27 days.

### 3.3. Intraoperative

RA and monitored anaesthetic care (MAC) were the predominant anaesthetic techniques of choice (Table 2). The majority of our patients received the standard ASA intraoperative monitoring. However, only 2 patients received intraoperative temperature monitoring. Bispectral index (BIS) monitoring was applied in slightly over a quarter of the patients receiving GA (26.7%).

Intraoperative hypotension was seen in 25.8 % of the patients, and it occurred exclusively in patients who were administered GA or spinal anaesthesia, with the exception of one case, which involved sedation for a lower limb angioplasty. Amongst this group who experienced hypotension, 2 had ischemic heart disease (IHD), 2 had previous strokes, and all except 2 patients had at least 1 cardiovascular risk factor. Only 56% of those with intraoperative hypotension received vasopressors in the form of phenylephrine or ephedrine boluses. All of these hypotensive patients recovered uneventfully.

### 3.4. Postoperative

The mean duration of surgery was 64 min (range 10–150 min). Hypothermia, desaturation, and hypertension (Table 3) were the top three complications observed in PACU. Five of our patients required rescue analgesia. Amongst these 5, 3 underwent GA for the following surgeries: rigid cystoscopy, open mesh inguinal hernia repair, and wide excision of buccal carcinoma with neck dissection. The other 2 received spinal anaesthesia for dynamic hip screw insertion. Over a quarter (27.4%) of patients were admitted to a high-dependency (HD) facility postoperatively and none to ICU. The average length of hospitalisation was 13.7 (range 1–56) days. 22.6% of the patients had their discharge delayed due to medical reasons, 21.3% due to social reasons, and in 8.2% delay was contributed by both. There were a total of 6 deaths, of which only 1 occurred within the 30-day postoperative period. This patient underwent a successful femoral embolectomy for critical limb ischemia. Postoperatively, the patient was discharged to a high-dependency unit. Apart from hypertension (systolic blood pressure of 170–180), there were no other immediate perioperative complications.

### 3.5. Hip Surgery Subgroup

Amongst the 62 patients, 36 underwent orthopaedic surgeries, of which 21 involved hip surgeries (Table 4). Majority of these patients (95%) were ASA 2, and 3.8% of the 21 patients were referred to CVM for preoperative risk stratification and optimisation. Approximately less than one-third received GA (28.6%), whilst the remaining patients received spinal anaesthesia with MAC. In the postoperative period, 2 out of 6 who received GA needed blood transfusion postoperatively, whereas only 1 out of 15 in the spinal anaesthesia group warranted a blood transfusion. The average length of hospital stay was noted to be slightly longer in the group that received GA (14.7 days) versus 13.9 days in the spinal group. There were no ICU admissions or 30-day mortalities in this subgroup.

## 4. Discussion

This is the first study in Singapore and Asia examining the anaesthesia outcomes in the extreme elderly. Our results resonate findings of previous studies [5] that these patients tend to do well postoperatively, with relatively low incidence of postoperative complications and 30-day mortality rates. Most of them are ASA 3 patients who are independent in their activities of daily living (ADL), undergoing intermediate cardiac risk surgeries. We postulate that one of the reasons for the good outcomes could be stringent patient selection by the surgeons and anaesthetists.

### 4.1. Preoperative Assessment and Optimisation

Preoperative assessment is especially important in the very elderly because of their multi-comorbidities, age-related physiological decline, and frailty. History and physical examination remains the cornerstone of preoperative assessment. Patients with abnormal clinical findings and/or unknown functional status should undergo further cardiac testing if they are planned for elective surgeries [6].

A recent retrospective cohort study showed that preoperative cardiac ultrasound performed by anaesthetists did not result in operative delay and is associated with decreased perioperative mortality in patients with raised cardiac risk factors [6]. One observational study supports the performance of anaesthetist-led preoperative cardiac ultrasound as it was found to have high correlation with formal TTE findings, and the findings altered anaesthetic management in 20.8% of their patients [7]. Current ACC/AHA guidelines recommend preoperative TTE in patients with valvular lesions, preexisting left ventricular dysfunction, or clinical evidence of heart failure. Anaesthetist-led intraoperative TEE is also recommended in patients with hemodynamic instability refractory to corrective therapy [6]. Our current hospital inpatient workflow mandates a cardiac referral as a prerequisite for TTE. Revising this practice by giving primary teams rights to order echocardiograph or moving towards the direction of anaesthetist-led bedside cardiac ultrasound can streamline the preoperative optimisation process. This will also minimize unnecessary referrals and delays in surgeries.

### 4.2. Anaesthesia and Monitoring

Intra-arterial BP monitoring was applied in only 3 patients who underwent the following surgeries: femoral embolectomy, laparotomy with repair of abdominal wall hernia, and trephine colostomy. The benefits of arterial cannulation, including its ability to monitor beat-to-beat arterial pressure variation and convenient blood sampling may be obvious, yet there is a paucity of studies looking at its impact on patient outcomes [8]. Gershengorn et al. did not find any mortality benefit in ICU patients receiving arterial cannulas [9]. Likewise, AAGBI 2014 guidelines did not provide any specific recommendations for the use of intra-arterial BP monitoring in the elderly, except that when arterial cannulas are used, they should be inserted and transduced before induction [10].

### 4.3. Haemodynamics

Intraoperative hypotension occurred in a quarter (25.8%) of our patients. Despite this, none of them suffered any cardiac or neurological sequelae during the perioperative period. In the one patient who experienced intraoperative hypotension with RA, it was likely due to a combination of fentanyl boluses, vancomycin, and dexmedetomidine infusion, given in close succession at induction. Evidence on the causal relationship between perioperative hypotension and cardiac events has been mixed. Some studies found a significant increase in postoperative cardiac events amongst patients who experienced a decrease in mean arterial pressure (MAP) of ≥20% or pulse rate increase of ≥20 beats/min [11–13], while in another study no difference was detected [14]. Nonetheless, the deleterious effects of relative hypotension in this elderly group should not be taken lightly as preexisting arteriolosclerosis and hypertension lowers the threshold for ischemia tolerance in the myocardial, cerebral, and renal tissues.

Hypovolemia secondary to blood loss could also contribute to the hypotension. Half of our patients who experienced intraoperative hypotension underwent a hip surgery where significant blood loss was likely. As such, close surveillance of the intraoperative blood loss and prompt fluid replacement therapy should be instituted in surgeries with anticipated blood loss.

### 4.4. Postoperative Complications and Mortalities

Despite the widespread use of warming devices, a significant proportion of our patients (20.7%) were hypothermic upon arrival in recovery. This finding is consistent with knowledge that the elderly have impaired heat conservation mechanisms [15]. The adverse outcomes of perioperative hypothermia have been well established: cardiac arrhythmias, coagulopathy resulting in postoperative bleeding and anaemia, delirium, poor wound healing, and prolonged hospital stay [16]. Normothermia has also been shown to decrease the risk of cardiac morbidity by 55%. In light of this, it is paramount that we consider more aggressive temperature conservation strategies in this susceptible group. For instance, practices such as routine intraoperative temperature monitoring, use of continuous fluid warmers, and plastic sheets to cover exposed areas should be reinforced.

Eleven patients (17.7%) were hypoxic in PACU. But all of their saturations improved to ≥95% upon oxygen supplementation (up to FiO_2_ 0.5). No further interventions were instituted, except in one patient where chest physiotherapy and suctioning were performed in the recovery by a respiratory therapist. None of them developed any respiratory complications subsequently, and all were successfully weaned off oxygen supplementation in the wards. Eight of the 11 desaturation cases involved GA or RA combined with MAC. Not surprisingly, patients who received GA or sedation were more likely to desaturate than those who received solely RA. Desaturation in this group can be attributed to atelectasis and hypoventilation due to residual anaesthetic effects.

Poorly controlled high BP has been repeatedly shown to be associated with increased perioperative morbidity and mortality [17]. A review article looking at 30 observational studies demonstrated an odds ratio of 1.35 for an association between hypertension and perioperative cardiac outcomes [18]. In our study, amongst the 6 patients who developed hypertension in PACU, all had preexisting hypertension. Two of these 6 were untreated in PACU. No subsequent complications in the form of cardiac or cerebrovascular events were observed in them. While the autoregulation curve is right-shifted in chronically hypertensive patients, BP of ≥180/110 constitutes a hypertensive crisis and should not be left untreated [19]. It is also important to consider other contributors to postoperative hypertension, such as pain and urinary retention, before initiating antihypertensive therapy.

Of the 6 who developed confusion, one already was delirious preoperatively, while another was readmitted for altered mental state 2 days later due to hyponatremia. In another patient, his history was significant for chronic alcoholism, a known risk factor for delirium. None of the 6 patients received benzodiazepine, ketamine, or anticholinergics perioperatively. Previously thought to be contributors to delirium in elderly, recent studies fail to show any association between these drugs and the development of postoperative delirium (POD) [10, 20–22]. One of the purported benefits of RA is a lower rate of POD compared to GA. However, no difference has been detected in terms of the incidence of long-term postoperative cognitive dysfunction (POCD) in the RA versus GA group [23]. Amongst our patients with delirium, 3 received RA, 2 received GA, and 1 received GA following a failed spinal. Hence, no conclusions can be drawn from our study on POD with respect to the anaesthetic technique.

The mortality case involved a patient who initially presented with urosepsis secondary to calculus obstructive uropathy. He received culture-guided antibiotics therapy and underwent a percutaneous nephrostomy procedure under local anaesthesia. His admission was further complicated by critical limb ischemia for which he underwent a successful femoral embolectomy within 24 hours of presentation. Postoperatively, the patient developed hypotension 4 hours after surgery, which degenerated to pulseless electrical activity collapse. The patient was successfully resuscitated; in the meantime, a do-not-resuscitate status was established with the family in view of the patient's advanced age and need for long-term dialysis. The patient eventually demised on the same day. The cause of death was ascertained by the coroner as urosepsis. In light of the recent major vascular surgery with its associated high cardiac risk, myocardial infarction is a likely contributory cause to death.

### 4.5. Hip Surgeries

Of the 21 hip surgeries performed, slightly over two-thirds (71.4%) were conducted under spinal with MAC. This preponderance for spinal anaesthesia reflects the preferred choice of anaesthetic amongst anaesthetists caring for this subset of patients. A systemic review of 141 trials revealed approximately a third reduction in incidence of overall mortality and myocardial infarction rates in the centraneuraxial group undergoing a variety of surgeries [24]. This group was also found to have reduced venothromboembolic events, pneumonia, respiratory depression, and transfusion requirements [25, 26]. In our experience, only 1 out of the 22 patients who underwent hip surgery suffered a DVT, and this patient received a GA. Our sample size, however, is too small to establish any associations. There were no significant differences seen between the 2 anaesthetic techniques with regard to the incidence of intraoperative and postoperative complications. It is, however, notable that the blood transfusion rate was higher in the GA group.

As with limitations inherent to all retrospective studies, medical records were not always complete and patients with such records were excluded. In addition, this study included elderly patients considered for surgery and tends to be healthier than general elderly population. Postoperative delirium was assessed clinically, but together with postoperative cognitive dysfunction were not formally screened for during the period of hospitalisation and after discharge. As such, their true incidence cannot be determined in our study. Lastly, due to the small sample size of our study and heterogeneity of patient factors and surgery type, we are unable to stratify patients according to their demographics, anaesthetic techniques, or types of surgeries to compare their outcomes or draw any meaningful conclusions (except for the subgroup of patients who underwent hip surgery).

## 5. Conclusion

This study provides a snapshot of the preoperative outcomes of the very elderly patients. In conclusion, the outcome of postanaesthesia and surgery was good in the very elderly population, evident in their relatively low postoperative complication, and 30-day mortality rates. This is in spite of their multiple comorbidities, reduced reserves, and the emergency nature of majority of these operations. Findings derived from this study will enhance our understanding towards extreme elderly undergoing operation, provide risk assessment, and help tailor our anaesthetic techniques to optimize their outcomes. Moving forward, screening for frailty could be incorporated in future preoperative anaesthetic optimisation process with the view of implementing prehabilitation regimens. Evidence exists that prehabilitation implemented in the elderly and frail improves postoperative outcomes [27].

## Figures and Tables

**Table 1:** Preoperative patient demographics and surgical characteristics (*n* = 62).

	No. of patients (%)
Gender	
Male	22 (35.5)
Female	40 (64.5)
ASA physical status	
1	2 (3.2)
2	16 (25.8)
3	44 (71.0)
ADL^*∗*^	
Independent	36 (67.9)
Assisted	17 (32.1)
Comorbidities	
Hypertension	43 (69.4)
Ischemic heart disease (IHD)	11 (17.7)
Arrhythmia	10 (16.1)
Chronic renal failure	5 (8.1)
End-stage renal failure	0 (0)
Diabetes mellitus	13 (21)
Stroke/TIA	8 (12.9)
COPD/asthma	3 (4.8)
Dementia	6 (9.7)
Anaemia^≠^	27 (43.5)
Preexisting coagulopathy	
Pharmacological	29 (46.8)
Nonpharmacological^*∗*^	14 (24.6)
Type of surgery	
Emergency	47 (75.8)
Elective	15 (24.2)
Severity of surgery	
Minor	19 (34.5)
Intermediate	32 (58.2)
Major	4 (7.3)
Surgical discipline	
Orthopaedic	36 (67.3)
Vascular	3 (5.5)
General surgery	10 (18.2)
Urology	5 (9.1)

ADL, activities of daily living. ≠Anaemia is defined in accordance with local haematological lab ranges (Hb: male <13; female <12). Emergency operations are defined as surgeries needing to be performed within 6 hours of diagnosis or establishment of surgical indication; elective surgeries are defined as those that can be delayed for more than 24 hours. We defined the severity of the operation (minor, intermediate, or major surgery) in accordance with the American College of Cardiology/American Heart Association (ACC/AHA) cardiac risk stratification; hypertension is defined as NIBP systolic ≥180 for 2 or more consecutive readings. ^*∗*^Missing data present, percentage taken as total available data.

**Table 2:** Intraoperative characteristics and events (*n* = 62).

	No. of patients (%)
Anaesthetic technique	
GA	18 (29.0)
RA	36 (58.1)
Spinal	27 (43.5)
Epidural	4 (6.5)
PNB	5 (8.1)
MAC	5 (8.1)
RA + MAC	3 (4.8)
Monitoring	
IA	3 (4.8)
CVP	0 (0)
BIS	4 (6.5)
Temperature	2 (3.2)
Intraoperative events	
Hypotension	16 (25.8)
Required vasopressor boluses	25 (40.3)
Desaturation	1 (1.6)
Blood transfusion	9 (14.5)

MAC, monitored anaesthesia care; RA, regional anaesthesia; GA, general anaesthesia. RA + MAC refers to patients receiving both regional anaesthesia (centraneuraxial block or PNB) and monitored anaesthetic care. Hypertension is defined as NIBP systolic ≥180 for 2 or more consecutive readings. PONV, postoperative nausea and vomiting. ^*∗*^Missing data present, percentage taken as total available data.

**Table 3:** Postoperative outcomes and complications (*n* = 62).

	No. of patients (%)
Rescue analgesia in PACU	5 (8.1)
Postoperative complications	
PACU	
Hypothermia^*∗*^	12 (20.7)
Desaturation	11 (17.7)
Hypertension	6 (9.7)
Hypotension (in PACU)	3 (4.8)
Tachyarrthymia	2 (3.2)
PONV	2 (3.2)
In the wards	
Delirium	6 (9.7)
Urinary tract infection	4 (6.5)
Sepsis	3 (4.8)
Bleeding (surgical site)	3 (4.8)
Wound infection	2 (3.2)
Pneumonia	2 (3.2)
Venothromboembolic event	1 (1.6)
30-day mortality	1 (1.6)
HD admission	17 (27.4)
ICU admission	0 (0)
Readmission	4 (6.5)

Readmissions are defined as repeat hospitalisation where the primary problem is related to the same surgical problem. Patient records are screened up to 6 months after discharge for readmissions.

**Table 4:** Hip surgeries (*n* = 21).

	No. of patients (%)	
ASA status		
1	1 (4.8)	
2	9 (42.9)	
3	11 (52.4)	

Anaesthetic technique	GA	Spinal

	6 (28.6)	15 (71.4)
Intraoperative complications		
Hypotension	2 (33.3)	5 (33.3)
PACU complications		
Desaturation	2 (33.3)	3 (20)
Hypertension	2 (33.3)	—
PONV	—	1 (6.7)
Postoperative complications		
Bleeding (surgical site)	1 (16.7)	1 (6.7)
Urinary tract infection	2 (33.3)	2 (13.3)
Venothromboembolic event	1 (16.7)	—
Arrthymia		1 (6.7)
Blood transfusion	2 (33.3)	1 (6.7)
Length of stay		
(Average, days)	14.7	13.9
30-day mortality	—	—

## Data Availability

The data used to support the findings of this study are included within the article.

## References

[1] Department of Statistics (2016). *Complete Life Tables 2008–2013 for Singapore Resident Population*.

[2] Fleisher L. A., Fleischmann K. E., Auerbach A. D. (2014). ACC/AHA guideline on perioperative cardiovascular evaluation and management of patients undergoing noncardiac surgery. *Journal of the American College of Cardiology*.

[3] American Society of Anesthesiologists (2016). *Standards and Practice Parameters: Standards for Basic Anesthetic Monitoring*.

[4] Aldrete J. A. (1995). The post-anesthesia recovery score revisited. *Journal of Clinical Anesthesia*.

[5] Konttinen N., Rosenberg P. H. (2006). Outcome after anaesthesia and emergency surgery in patients over 100 years old. *Acta Anaesthesiologica Scandinavica*.

[6] Canty D. J., Royse C. F., Kilpatrick D., Bowyer A. (2012). The impact on cardiac diagnosis and mortality of focused transthoracic echocardiography in hip fracture surgery patients with increased risk of cardiac disease: a retrospective cohort study. *Anesthesia*.

[7] Andruszkiewicz P., Sobczyk D., Gorkiewicz-Kot I., Kowalik I., Gelo R., Stach O. (2015). Reliability of focused cardiac ultrasound by novice sonographer in preoperative anaesthetic assessment: an observational study. *Cardiovascular Ultrasound*.

[8] Gardland A. (2001). Arterial lines in the ICU. *Chest*.

[9] Gershengorn H. B., Wunsch H., Scales D. C. (2014). Association between arterial catheter use and hospital mortality in intensive care units. *JAMA Internal Medicine*.

[10] Griffiths R., Beech F., Brown A. (2014). Peri-operative care of the elderly 2014. *Anaesthesia*.

[11] Charlson M. E., Mackenzie C. R., Gold J. P. (1989). The preoperative and intraoperative hemodynamic predictors of postoperative myocardial infarction or ischemia in patients undergoing noncardiac surgery. *Annals of Surgery*.

[12] Shah K. B., Kleinman B. S., Sami H., Pate J., Rao T. L. (1990). Re-evaluation of perioperative myocardial infarction in patients with prior myocardial infarction undergoing noncardiac operations. *Anesthesia & Analgesia*.

[13] Williams-Russo P., Sharrock N. E., Mattis S. (1999). Randomised trial of hypotensive epidural anesthesia in older adults. *Anesthesiology*.

[14] Badner N. H., Knill R. L., Brown J. E., Novick T. V., Gelb A. W. (1998). Myocardial infarction after noncardiac surgery. *Anaesthesiology*.

[15] Van de Kerkhof P. C., Van Bergen B., Spruijt K., Kuiper J. P. (1994). Age related changes in wound healing. *Clinical and Experimental Dermatology*.

[16] Reynolds L., Beckman J., Kurz A. (2008). Perioperative complications of hypothermia. *Best practice and research, Clinical Anaesthesiology*.

[17] Howell S. J., Hemming A. E., Allman K. G., Glover L., Sear J. W., Foex P. (1997). Predictors of postoperative myocardial ischemia. The role of intercurrent arterial hypertension and other cardiovascular risk factors. *Anaesthesia*.

[18] Howell S. J., Sear J. W., Foex P. (2004). Hypertension, hypertensive heart disease and perioperative cardiac risk. *British Journal of Anaesthesia*.

[19] Varon J., Marik P. E. (2008). Perioperative hypertension management. *Vasc Health Risk Management*.

[20] Williams-Russo P., Sharrock N. E., Mattis S., Szatrowski T. P., Charlson M. E. (1995). Cognitive effects after epidural vs general anaesthesia in older adults a randomised trial. *JAMA*.

[21] Moller J. Y., Cluitmans P., Rasmussen L. S. (1998). Long term postoperative cognitive dysfunction in the elderly ISPOCD 1 study. ISPOCD investigators. International study of post-operative cognitive dysfunction. *Lancet*.

[22] Rasmussen L. S., Steentoft A., Rasmussen H., Kristensen P. A., Moller J. T. (1999). Benzodiazepines and postoperative cognitive dysfunction in the elderly. ISPOCD group. International study of postoperative cognitive dysfunction. *British Journal of Anaesthesia*.

[23] Rasmussen L. S., Johnson T., Kuipers H. M. (2003). Does anesthesia cause post operative cognitive dysfunction: a randomised study of regional versus general anaesthesia in 438 elderly patients. *Acta Anaesthesiologica Scandinavica*.

[24] Rogers A., Walker N., Schug S. (2000). Reduction of postoperative mortality and morbidity with epidural or spinal anaesthesia: results from overview of randomised trials. *BMJ*.

[25] Dauphin A., Raymer K. E., Stanton E. B., Fuller H. D. (1997). Comparison of general anesthesia with and without lumbar epidural for total hip arthroplasty: effects of epidural block on hip arthroplasty. *Journal of Clinical Anesthesia*.

[26] Urwin S. C., Parker M. H., Griffiths R. (2000). General versus regional anesthesia for hip fracture surgery: a meta-analysis of randomized trials. *British Journal of Anaesthesia*.

[27] Souwer E. T. D., Bastiaannet E., Brujin S. D. (2018). Comprehensive multidisciplinary care program for elderly colorectal cancer patients: “from prehabilitation to independence”. *European Journal of Surgical Oncology*.

